# *catena*-Poly[[[*cis*-aqua­dibromido­cobalt(II)]-μ-(pyrazine-2-carb­oxy­lic acid)-κ^3^ *N* ^1^,*O*:*N* ^4^] monohydrate]

**DOI:** 10.1107/S1600536811048628

**Published:** 2011-11-23

**Authors:** Christopher Dares, Rene Fournier, A. B. P. Lever

**Affiliations:** a Department of Chemistry, York University, Toronto, Ontario, Canada M3J 1P3

## Abstract

The title compound, {[CoBr_2_(C_5_H_4_N_2_O_2_)(H_2_O)]·H_2_O}_*n*_, is a one-dimensional coordination polymer which crystallizes as a monohydrate. The asymmetric unit contains one Co^II^ atom in a distorted octa­hedral geometry, forming a chain parallel to [010] with the pyrazine carb­oxy­lic acid ligands coordinating on one side in a bidentate fashion through one N and one O atom, and in a monodentate fashion through a N atom, with N atoms *trans*, and with both ligands lying in the same plane. The bromide atoms are *cis* to each other, while a water mol­ecule occupies the final octa­hedral coordination site. The chains are linked together though an O—H⋯Br hydrogen bonding network, and are further stabilized by an O—H⋯Br and O—H⋯O hydrogen-bonding framework with the solvent water mol­ecule.

## Related literature

For the synthesis of related compounds, see: Gao *et al.* (2007 ▶) and references therein. For other examples of linear coordin­ation polymers utilizing pyrazine derivatives, see: Mao *et al.* (1996 ▶). 
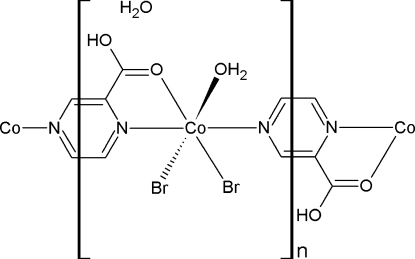


## Experimental

### 

#### Crystal data

[CoBr_2_(C_5_H_4_N_2_O_2_)(H_2_O)]·H_2_O
*M* *_r_* = 378.88
Monoclinic, 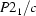

*a* = 6.9367 (3) Å
*b* = 13.9983 (3) Å
*c* = 11.1446 (5) Å
β = 106.043 (2)°
*V* = 1040.02 (7) Å^3^
*Z* = 4
Mo *K*α radiation
μ = 9.32 mm^−1^
*T* = 150 K
0.18 × 0.16 × 0.06 mm

#### Data collection

Bruker–Nonius KappaCCD diffractometer
Absorption correction: multi-scan (*SORTAV*; Blessing, 1995 ▶) *T*_min_ = 0.399, *T*_max_ = 0.962
7275 measured reflections
2375 independent reflections
2013 reflections with *I* > 2σ(*I*)
*R*_int_ = 0.055

#### Refinement

*R*[*F* ^2^ > 2σ(*F* ^2^)] = 0.036
*wR*(*F* ^2^) = 0.088
*S* = 1.04
2375 reflections
147 parameters
1 restraint
H atoms treated by a mixture of independent and constrained refinement
Δρ_max_ = 0.77 e Å^−3^
Δρ_min_ = −1.25 e Å^−3^

### 

Data collection: *COLLECT* (Nonius, 2002 ▶); cell refinement: *DENZO-SMN* (Otwinowski & Minor, 1997 ▶); data reduction: *DENZO-SMN*; program(s) used to solve structure: *SHELXS97* (Sheldrick, 2008 ▶); program(s) used to refine structure: *SHELXL97* (Sheldrick, 2008 ▶); molecular graphics: *ORTEP-3 for Windows* (Farrugia, 1997 ▶); software used to prepare material for publication: *WinGX* (Farrugia, 1999 ▶).

## Supplementary Material

Crystal structure: contains datablock(s) global, I. DOI: 10.1107/S1600536811048628/bx2383sup1.cif

Structure factors: contains datablock(s) I. DOI: 10.1107/S1600536811048628/bx2383Isup2.hkl

Additional supplementary materials:  crystallographic information; 3D view; checkCIF report

## Figures and Tables

**Table 1:** Selected geometric parameters (Å, °)

Co1—O1	2.073 (3)
Co1—N1	2.139 (3)
Co1—N2	2.179 (3)
Co1—O2	2.185 (2)
Co1—Br1	2.5499 (6)
Co1—Br2	2.5522 (6)

**Table 2:** Hydrogen-bond geometry (Å, °)

*D*—H⋯*A*	*D*—H	H⋯*A*	*D*⋯*A*	*D*—H⋯*A*
O1—H1*A*⋯Br1^i^	0.91 (5)	2.31 (5)	3.212 (3)	169 (4)
O1—H1*B*⋯Br2^ii^	0.87 (5)	2.39 (5)	3.251 (3)	173 (5)
O1*W*—H1*W*⋯Br1^iii^	0.75 (6)	2.68 (6)	3.390 (3)	159 (6)
O1*W*—H2*W*⋯Br2^iv^	0.76 (6)	2.57 (6)	3.335 (4)	176 (7)
O3—H3*W*⋯O1*W*	0.81 (5)	1.76 (5)	2.543 (5)	166 (6)

Symmetry codes: (i) 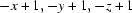; (ii) 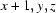; (iii) 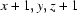; (iv) 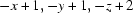.

**Fig. 1.**
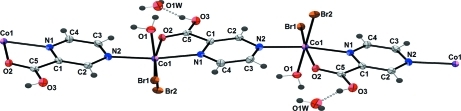
A view of (I) with atom numbering scheme showing the molecular structure and intra- and intermolecular H bonding present. Displacement ellipsoids are drawn at the 50% probability level.

**Fig. 2.**
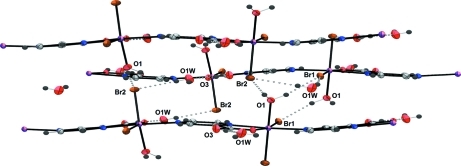
Crystal packing diagram of (I) showing layers of (I) interacting via the H bonding network. Displacement ellipsoids are drawn at the 50% probability level.

## supplementary crystallographic information

### Comment

The title compound (I) forms a linear 1-D coordination polymer aligned along *b*, with pyrazine carboxylic acid ligands linking Co^II^ metal centres together in a bidentate fashion to one cobalt through N and O atoms, and in a monodentate fashion through the remaining N atom, with N atoms *trans* to each other, and neighboring pyrazine rings within the same plane. The two bromide anions are coordinated in a *cis* arrangement, with a water molecule completing the distorted octahedral geometry about the Co^II^. The asymmetric unit includes only a single monomer, with the 2_1_ screw axis generating the neighboring 'inverted' linked monomer.The Co–N bonds average 2.16 Å, while the Co–O_pz_ bond length is 2.18 Å. The Co–Br bonds are essentially identical at 2.55 Å.

Linear chains directly interact with each other through hydrogen bonding between the coordinated water, and bromide ligands. The single water solvate is involved heavily in the hydrogen bonding network interacting with both bromide anions, as well as the carboxylic acid group further stabilizing the crystal structure.

### Experimental

In a synthesis designed to form *mer*-tris(pyrazine carboxylato)cobalt(III), CoBr_2_^.^6(H_2_O) was dissolved in methanol at room temperature to which three equivalents of pyrazine carboxylic acid was added. The initial red precipitate that formed almost immediately and was identified as *mer*-tris(pyrazine carboxylato)cobalt(III) bromide was removed by filtration. To the mother liquor was added an equal volume of water. Subsequently, the blue solution was allowed to stand for 2 months at room temperature allowing (I) to crystallize by slow evaporation yielding bright pink prismatic crystals suitable for X-ray diffraction. Attempts to remake (I) *via* more rational routes using CoBr_2_^.^6(H_2_O) and one equivalent of pyrazine carboxylic acid were not successful.

### Refinement

All H atoms attached to C atoms were added in ideal locations, and constrained to ride on the parent atoms with *U*_iso_ = 1.2*U*_eq_(C). The H atoms attached to O atoms were located in the electron density difference map, and, with the exception of H1B were allowed to refine spatially and thermally. H1B was restrained to be 0.82 ± 0.02 Å from O1.

### Crystal data

**Table:**

[CoBr_2_(C_5_H_4_N_2_O_2_)(H_2_O)]·H_2_O	*F*(000) = 724
*M**_r_* = 378.88	*D*_x_ = 2.42 Mg m^−^^3^
Monoclinic, *P*2_1_/*c*	Mo *K*α radiation, λ = 0.71073 Å
Hall symbol: -P 2ybc	Cell parameters from 3286 reflections
*a* = 6.9367 (3) Å	θ = 2.6–27.5°
*b* = 13.9983 (3) Å	µ = 9.32 mm^−^^1^
*c* = 11.1446 (5) Å	*T* = 150 K
β = 106.043 (2)°	Prism, pink
*V* = 1040.02 (7) Å^3^	0.18 × 0.16 × 0.06 mm
*Z* = 4	

### Data collection

**Table:**

Bruker–Nonius KappaCCD diffractometer	2375 independent reflections
Radiation source: fine-focus sealed tube	2013 reflections with *I* > 2σ(*I*)
graphite	*R*_int_ = 0.055
φ scans and ω scans with κ offsets	θ_max_ = 27.5°, θ_min_ = 2.9°
Absorption correction: multi-scan (*SORTAV*; Blessing, 1995)	*h* = −8→8
*T*_min_ = 0.399, *T*_max_ = 0.962	*k* = −17→18
7275 measured reflections	*l* = −14→14

### Refinement

**Table:**

Refinement on *F*^2^	Primary atom site location: structure-invariant direct methods
Least-squares matrix: full	Secondary atom site location: difference Fourier map
*R*[*F*^2^ > 2σ(*F*^2^)] = 0.036	Hydrogen site location: inferred from neighbouring sites
*wR*(*F*^2^) = 0.088	H atoms treated by a mixture of independent and constrained refinement
*S* = 1.04	*w* = 1/[σ^2^(*F*_o_^2^) + (0.051*P*)^2^] where *P* = (*F*_o_^2^ + 2*F*_c_^2^)/3
2375 reflections	(Δ/σ)_max_ < 0.001
147 parameters	Δρ_max_ = 0.77 e Å^−^^3^
1 restraint	Δρ_min_ = −1.25 e Å^−^^3^

### Special details

**Table:**

Experimental. multi-scan from symmetry-related measurements Sortav (Blessing 1995)
Geometry. All s.u.'s (except the s.u. in the dihedral angle between two l.s. planes) are estimated using the full covariance matrix. The cell s.u.'s are taken into account individually in the estimation of s.u.'s in distances, angles and torsion angles; correlations between s.u.'s in cell parameters are only used when they are defined by crystal symmetry. An approximate (isotropic) treatment of cell s.u.'s is used for estimating s.u.'s involving l.s. planes.
Refinement. Refinement of *F*^2^ against ALL reflections. The weighted *R*-factor *wR* and goodness of fit *S* are based on *F*^2^, conventional *R*-factors *R* are based on *F*, with *F* set to zero for negative *F*^2^. The threshold expression of *F*^2^ > 2σ(*F*^2^) is used only for calculating *R*-factors(gt) *etc*. and is not relevant to the choice of reflections for refinement. *R*-factors based on *F*^2^ are statistically about twice as large as those based on *F*, and *R*- factors based on ALL data will be even larger.

### Fractional atomic coordinates and isotropic or equivalent isotropic displacement parameters (Å^2^)

**Table:**

	*x*	*y*	*z*	*U*_iso_*/*U*_eq_	
Co1	0.43042 (7)	0.60139 (3)	0.71677 (4)	0.01247 (14)	
Br1	0.21778 (6)	0.59962 (2)	0.49002 (3)	0.01792 (13)	
Br2	0.14725 (6)	0.61738 (3)	0.82087 (3)	0.02000 (13)	
O1	0.6859 (4)	0.5836 (2)	0.6571 (3)	0.0203 (6)	
O2	0.6433 (4)	0.62621 (18)	0.8992 (2)	0.0154 (5)	
O3	0.8202 (4)	0.7413 (2)	1.0224 (2)	0.0212 (6)	
N1	0.4772 (4)	0.7525 (2)	0.7222 (3)	0.0138 (6)	
N2	0.4431 (4)	0.4469 (2)	0.7415 (3)	0.0141 (6)	
C1	0.6070 (5)	0.7858 (2)	0.8275 (3)	0.0135 (7)	
C2	0.3514 (5)	0.3823 (3)	0.6545 (3)	0.0140 (7)	
H2	0.2574	0.4034	0.5799	0.017*	
C3	0.5688 (6)	0.4125 (3)	0.8461 (3)	0.0169 (8)	
H3	0.6336	0.4558	0.9101	0.020*	
C4	0.3927 (6)	0.8159 (3)	0.6360 (3)	0.0161 (7)	
H4	0.3032	0.7946	0.5599	0.019*	
C5	0.6938 (5)	0.7099 (3)	0.9208 (3)	0.0136 (7)	
H1A	0.701 (7)	0.527 (4)	0.619 (5)	0.040 (14)*	
H1B	0.802 (5)	0.597 (4)	0.700 (6)	0.07 (2)*	
H3W	0.854 (7)	0.695 (4)	1.066 (5)	0.035 (14)*	
O1W	0.9753 (5)	0.6131 (3)	1.1815 (3)	0.0282 (7)	
H1W	1.020 (9)	0.625 (4)	1.249 (6)	0.038 (17)*	
H2W	0.952 (7)	0.560 (4)	1.184 (5)	0.035 (15)*	

### Atomic displacement parameters (Å^2^)

**Table:**

	*U*^11^	*U*^22^	*U*^33^	*U*^12^	*U*^13^	*U*^23^
Co1	0.0156 (3)	0.0113 (3)	0.0094 (3)	0.00003 (17)	0.0017 (2)	−0.00028 (17)
Br1	0.0230 (2)	0.0170 (2)	0.0106 (2)	0.00181 (13)	−0.00064 (16)	−0.00216 (12)
Br2	0.0186 (2)	0.0276 (2)	0.0140 (2)	−0.00205 (14)	0.00479 (16)	−0.00451 (14)
O1	0.0186 (16)	0.0208 (15)	0.0217 (16)	−0.0007 (11)	0.0061 (13)	−0.0048 (11)
O2	0.0205 (14)	0.0127 (13)	0.0113 (13)	−0.0008 (10)	0.0013 (11)	−0.0011 (9)
O3	0.0286 (16)	0.0173 (15)	0.0122 (13)	−0.0009 (11)	−0.0035 (12)	0.0001 (11)
N1	0.0162 (16)	0.0138 (15)	0.0127 (14)	−0.0007 (12)	0.0061 (13)	−0.0010 (12)
N2	0.0212 (17)	0.0133 (15)	0.0093 (14)	−0.0011 (12)	0.0064 (13)	0.0001 (11)
C1	0.0177 (19)	0.0142 (18)	0.0092 (17)	0.0019 (14)	0.0045 (15)	−0.0010 (13)
C2	0.016 (2)	0.0163 (18)	0.0100 (17)	−0.0005 (13)	0.0039 (15)	0.0013 (13)
C3	0.021 (2)	0.0177 (19)	0.0112 (18)	−0.0041 (15)	0.0034 (16)	−0.0028 (14)
C4	0.021 (2)	0.0163 (19)	0.0093 (16)	−0.0004 (14)	0.0014 (15)	−0.0018 (14)
C5	0.0141 (19)	0.019 (2)	0.0086 (16)	0.0027 (14)	0.0042 (14)	−0.0010 (13)
O1W	0.037 (2)	0.0257 (19)	0.0166 (17)	−0.0032 (14)	−0.0020 (15)	0.0060 (13)

### Geometric parameters (Å, °)

**Table:**

Co1—O1	2.073 (3)	N1—C1	1.349 (4)
Co1—N1	2.139 (3)	N2—C3	1.337 (5)
Co1—N2	2.179 (3)	N2—C2	1.350 (5)
Co1—O2	2.185 (2)	C1—C2^i^	1.384 (5)
Co1—Br1	2.5499 (6)	C1—C5	1.493 (5)
Co1—Br2	2.5522 (6)	C2—H2	0.9500
O1—H1A	0.92 (5)	C3—C4^ii^	1.382 (5)
O1—H1B	0.84 (2)	C3—H3	0.9500
O2—C5	1.227 (4)	C4—H4	0.9500
O3—C5	1.302 (4)	O1W—H1W	0.75 (6)
O3—H3W	0.80 (5)	O1W—H2W	0.77 (6)
N1—C4	1.320 (5)		
			
O1—Co1—N1	89.51 (11)	C4—N1—Co1	127.6 (2)
O1—Co1—N2	85.02 (11)	C1—N1—Co1	115.2 (2)
N1—Co1—N2	167.90 (12)	C3—N2—C2	116.7 (3)
O1—Co1—O2	84.25 (10)	C3—N2—Co1	117.5 (2)
N1—Co1—O2	76.01 (10)	C2—N2—Co1	125.4 (2)
N2—Co1—O2	92.67 (10)	N1—C1—C2^i^	121.7 (3)
O1—Co1—Br1	89.57 (8)	N1—C1—C5	113.8 (3)
N1—Co1—Br1	94.55 (8)	C2^i^—C1—C5	124.5 (3)
N2—Co1—Br1	96.20 (8)	N2—C2—C1^ii^	120.7 (3)
O2—Co1—Br1	168.71 (7)	N2—C2—H2	119.6
O1—Co1—Br2	171.91 (8)	C1^ii^—C2—H2	119.6
N1—Co1—Br2	91.74 (8)	N2—C3—C4^ii^	122.2 (3)
N2—Co1—Br2	92.21 (8)	N2—C3—H3	118.9
O2—Co1—Br2	88.30 (7)	C4^ii^—C3—H3	118.9
Br1—Co1—Br2	98.29 (2)	N1—C4—C3^i^	121.5 (3)
Co1—O1—H1A	118 (3)	N1—C4—H4	119.2
Co1—O1—H1B	125 (5)	C3^i^—C4—H4	119.2
H1A—O1—H1B	104 (5)	O2—C5—O3	125.4 (3)
C5—O2—Co1	114.6 (2)	O2—C5—C1	120.3 (3)
C5—O3—H3W	106 (4)	O3—C5—C1	114.2 (3)
C4—N1—C1	117.1 (3)	H1W—O1W—H2W	102 (5)

Symmetry codes: (i) −*x*+1, *y*+1/2, −*z*+3/2; (ii) −*x*+1, *y*−1/2, −*z*+3/2.

### Hydrogen-bond geometry (Å, °)

**Table:**

*D*—H···*A*	*D*—H	H···*A*	*D*···*A*	*D*—H···*A*
O1—H1A···Br1^iii^	0.91 (5)	2.31 (5)	3.212 (3)	169 (4)
O1—H1B···Br2^iv^	0.87 (5)	2.39 (5)	3.251 (3)	173 (5)
O1W—H1W···Br1^v^	0.75 (6)	2.68 (6)	3.390 (3)	159 (6)
O1W—H2W···Br2^vi^	0.76 (6)	2.57 (6)	3.335 (4)	176 (7)
O3—H3W···O1W	0.81 (5)	1.76 (5)	2.543 (5)	166 (6)

Symmetry codes: (iii) −*x*+1, −*y*+1, −*z*+1; (iv) *x*+1, *y*, *z*; (v) *x*+1, *y*, *z*+1; (vi) −*x*+1, −*y*+1, −*z*+2.
